# Conditional Deletion of Jak2 Reveals an Essential Role in Hematopoiesis throughout Mouse Ontogeny: Implications for Jak2 Inhibition in Humans

**DOI:** 10.1371/journal.pone.0059675

**Published:** 2013-03-27

**Authors:** Sung O. Park, Heather L. Wamsley, Kyungmi Bae, Zhongbo Hu, Xiaomiao Li, Se-woon Choe, William B. Slayton, S. Paul Oh, Kay-Uwe Wagner, Peter P. Sayeski

**Affiliations:** 1 Department of Physiology and Functional Genomics, University of Florida College of Medicine, Gainesville, Florida, United States of America; 2 Department of Physiological Sciences, University of Florida College of Veterinary Medicine, Gainesville, Florida, United States of America; 3 Department of Urology, University of Florida College of Medicine, Gainesville, Florida, United States of America; 4 Department of Pediatrics, University of Florida College of Medicine, Gainesville, Florida, United States of America; 5 Eppley Institute for Research in Cancer and Allied Diseases and Department of Pathology and Microbiology, University of Nebraska Medical Center, Omaha, Nebraska, United States of America; Northwestern University, United States of America

## Abstract

Germline deletion of Jak2 in mice results in embryonic lethality at E12.5 due to impaired hematopoiesis. However, the role that Jak2 might play in late gestation and postnatal life is unknown. To understand this, we utilized a conditional knockout approach that allowed for the deletion of Jak2 at various stages of prenatal and postnatal life. Specifically, Jak2 was deleted beginning at either mid/late gestation (E12.5), at postnatal day 4 (PN4), or at ∼2 months of age. Deletion of Jak2 beginning at E12.5 resulted in embryonic death characterized by a lack of hematopoiesis. Deletion beginning at PN4 was also lethal due to a lack of erythropoiesis. Deletion of Jak2 in young adults was characterized by blood cytopenias, abnormal erythrocyte morphology, decreased marrow hematopoietic potential, and splenic atrophy. However, death was observed in only 20% of the mutants. Further analysis of these mice suggested that the increased survivability was due to an incomplete deletion of Jak2 and subsequent re-population of Jak2 expressing cells, as conditional deletion in mice having one floxed Jak2 allele and one null allele resulted in a more severe phenotype and subsequent death of all animals. We found that the deletion of Jak2 in the young adults had a differential effect on hematopoietic lineages; specifically, conditional Jak2 deletion in young adults severely impaired erythropoiesis and thrombopoiesis, modestly affected granulopoiesis and monocytopoiesis, and had no effect on lymphopoiesis. Interestingly, while the hematopoietic organs of these mutant animals were severely affected by the deletion of Jak2, we found that the hearts, kidneys, lungs, and brains of these same mice were histologically normal. From this, we conclude that Jak2 plays an essential and non-redundant role in hematopoiesis during both prenatal and postnatal life and this has direct implications regarding the inhibition of Jak2 in humans.

## Introduction

Hematopoiesis is the process whereby hematopoietic stem cells in the bone marrow give rise to the terminally differentiated cells in the peripheral blood. The process is exquisitely controlled by a number of cytokines including granulocyte-macrophage colony-stimulating factor (GM-CSF), granulocyte colony-stimulating factor (G-CSF), macrophage colony-stimulating factor (M-CSF), erythropoietin (EPO), and thrombopoietin (TPO) to name a few. The binding of these cytokines to their cognate receptors on hematopoietic cells results in the activation of at least ten different *Src* family kinases and all four *Janus* family kinases within these cell types [1]–[4]. These activated kinases then phosphorylate a number of different intracellular substrates resulting in appropriate cell proliferation, differentiation, and subsequent hematopoiesis.

Janus kinase 2 (Jak2) is a member of the *Janus* family of tyrosine kinases. It was cloned in 1992 and found to be ubiquitously expressed in a number of animal tissues including hematopoietic organs [5]. Early signaling studies found Jak2 to be a critical mediator of both growth hormone and erythropoietin-dependent signaling [6], [7]. The *in vivo* importance of Jak2 in cytokine-dependent signaling was confirmed several years later when germline deletion of Jak2 in mice resulted in embryonic lethality by day 12.5 (E12.5) due to a lack of hematopoiesis [8], [9]. Despite the large number of kinases that are activated during hematopoiesis, these results indicated that at least during early embryonic development, there is no redundancy for the functional loss of Jak2. However, what role, if any, that Jak2 might play in hematopoiesis during the later stages of embryonic development, as well as in postnatal life, has not been previously explored.

In 2005, several groups independently reported a valine to phenylalanine substitution mutation at amino acid 617 of Jak2, in a large percentage of myeloproliferative neoplasm (MPN) patients [10]–[14]. MPNs are a group of heterogeneous diseases arising from a transformed hematopoietic stem cell and characterized by excessive numbers of one or more terminally differentiated blood cells of the myeloid lineage such as erythrocytes, thrombocytes, or white blood cells. The V617F mutation occurs somatically and leads to constitutive Jak2-dependent signaling in the absence of cytokine and hence, subsequent myeloid neoplasia. As a consequence of this, great effort has been made to identify Jak2 small molecule inhibitors for the treatment of MPNs. The hope is that these drugs can produce disease remission similar to that observed with tyrosine kinase inhibitor therapy for BCR/ABL chronic myeloid leukemia. While first generation Jak2 inhibitors have provided palliative relief for some disease associated symptomologies, they lack bone marrow efficacy in the form of histopathologic, cytogenetic, or molecular remissions [15]–[18], and as such, their impact on specific bone marrow progenitors is not well defined. Moreover, it is only within the past few years that the efficacy, toxicity, and suitability of Jak2 inhibitors has been evaluated in clinical trials, so the long term effects of Jak2 inhibition in humans are unknown [19]. This is an important consideration as Jak2 is expressed in nearly every tissue in the body and, in addition to hematopoiesis, it has been implicated in a number of other physiological and patho-physiological processes [20].

Here, we hypothesized that Jak2 plays a critical and non-redundant role in hematopoiesis throughout mouse ontogeny. To test this, we created a Jak2 conditional knockout (cKO) mouse that allowed for the temporal deletion of Jak2 during any stage of mouse ontogeny. We found that deletion of Jak2 beginning at either mid/late gestation (E12.5), postnatal day 4 (PN4), or ∼2 months of age resulted in severe hematopoietic insufficiency and subsequent death. In the adult animals, the specific cause of death was severe anemia and thrombocytopenia. From this, we conclude that Jak2 plays an essential and non-redundant role in hematopoiesis during prenatal and postnatal life and this has direct implications regarding the inhibition of Jak2 in humans.

## Materials and Methods

### Generation of Germline and Tamoxifen-inducible Deletion of Jak2 in Mice

**Figure 1:**
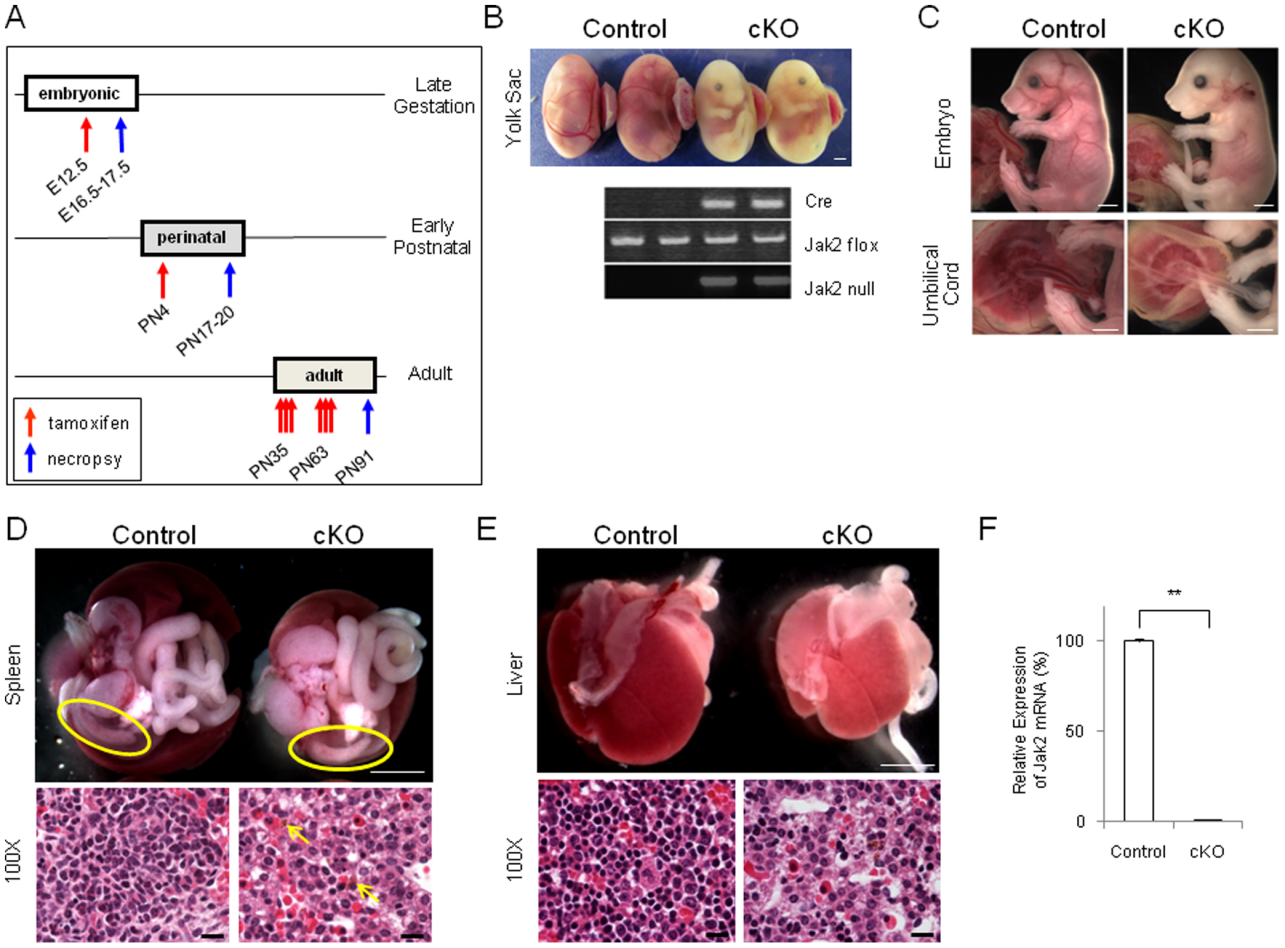
Deletion of Jak2 during mid/late gestation results in impaired erythropoiesis. (A) Cartoon illustrating the times at which Jak2 was deleted from mice via Tamoxifen injection and the times at which necropsy was performed. The three selected times for Jak2 deletion are mid/late gestation (E12.5), early prenatal (PN4), and early adult (PN35/PN63). Also shown are the times when necropsy was performed on each group; E16.5–E17.5, PN17–PN20, and PN91, respectively. (B**)** Representative control and Jak2 cKO E16.5 yolk sacs are shown as well as the PCR analysis that confirmed both the presence of Cre and the null allele in the Jak2 cKO embryos (n = 8), but not the controls (n = 12). Size bar  = 2 mm. (C) E17.5 embryos connected to visceral yolk sacs (top). Blood-filled umbilical vessels were seen in the control embryos, but not the Jak2 cKO embryos (bottom). Size bars  = 2 mm. (D) When compared to controls, spleens from the Jak2 cKO mice were paler (top) with visible histopathological necrosis (bottom, indicated by arrows). Upper panel size bar  = 2 mm while lower panel size bars  = 10 µm. (E) While livers from the control embryos appeared normal, livers from the Jak2 cKO embryos were similarly smaller and paler (top) and histopathology of E17.5 embryonic livers showed marked hypo-cellularity and reduced erythroid and megakaryocytic precursors in the Jak2 cKO mice (bottom). Upper panel size bar  = 2 mm while lower panel size bars  = 10 µm. (F) Determination of Jak2 mRNA levels in the liver from both genotypes (n = 3 for each genotype). **, p<0.01.

Mice were maintained under standard specific-pathogen-free conditions, and all animal procedures performed were reviewed and approved by the University of Florida Institutional Animal Care and Use Committee. The creation and characterization of the floxed Jak2 mice (*Jak2*) has been previously reported [21]. The germline Cre mice (Msx2-Cre), and the ROSA26-LacZ reporter mice have also been described [22], [23]. For inducible Jak2 deletion, Tamoxifen was dissolved in corn oil and ROSA26^CreER/+^;*Jak2* ^f/f^ (experimental) and ROSA26^+/+^;*Jak2* ^f/f^ (control) mice were injected intraperitoneally using a dosage of 1.25 mg/25 gram of body weight/day; injection days are shown on Figure 1A.

Genotypes were identified by polymerase chain reaction (PCR) with following specific primers: the *Cre* allele 5′-GCTAAACATGCTTCATCGTCGGTC and 5′-CAGATTACGTATATCCTGGCAGCG; *Jak2* floxed allele 5′-ATTCTGAGATTCAGGTCTGAGC and 5′-CTCACAACCATCTGTATCTCAC; *Jak2* null allele 5′-GTCTATACACCACCACTCCTG and 5′- AGCTGGAAAGATAGGTCAGC. For PCR reactions, after initial incubation at 94°C for 10 minutes, *Taq* polymerase was added to the reactions which were then amplified during 34 cycles of 94°C for 30 seconds, 60°C for 40 seconds, and 72°C for 40 seconds. Reaction products were separated and visualized on 2% agarose gels.

### Histologic Analysis

Mouse livers, spleens, kidneys, brains, hearts, and femurs were fixed overnight in buffered formalin and femurs were decalcified for 16 hours. Tissues were subsequently dehydrated through graded alcohols, paraffin embedded, sectioned at 5 µm, and stained with hematoxylin and eosin (H&E). Sections were examined under a light microscope for cellularity, necrosis, fibrosis, hemorrhage, and other lesions.

### Analysis of Peripheral Blood Samples

Mouse peripheral blood samples were obtained via submandibular bleeding. Complete blood counts (CBC) were determined by a hematology analyzer (HESKA). All readings were validated by a veterinary pathologist using peripheral blood films that were prepared in parallel and stained with Dip Quick (Jorgensen Laboratories). For the longitudinal studies, a small drop (∼5 µl) of blood was also applied to a HemoPoint H2 analyzer (Stanbio Life Sciences) to measure hemoglobin concentrations.

### Isolation of Bone Marrow

Bone marrow cells were prepared as previously described [24]. Briefly, femurs were flushed with 2 mL Dulbecco’s modified minimum essential medium using a 5 cc syringe fitted with a 27-gauge needle. Single cell suspensions were then prepared by repeatedly passing the harvested bone marrow cells through the needle. After lysis of red blood cells, bone marrow cells were either immediately frozen in liquid nitrogen and stored at –80°C for subsequent analysis or used directly for flow cytometry as described below.

### Real-time PCR

RNA was isolated from liver and bone marrow cells using the RNeasy Mini Kit (QIAGEN) and DNA contamination was removed using RNase-free DNase. cDNA was synthesized from 2 µg mRNA using the high capacity cDNA Reverse Transcription Kit (Applied Biosystems). Real-time PCR was performed on cDNA templates with a Multicolor Real-Time PCR Detection System using TaqMan gene expression assays (Applied Biosystems). PCR amplifications were performed in triplicate for Jak2 (Mm00434577_m1), GBP-2 (Mm00494576_g1), and IRF-1 (Mm01288580_m1) along with parallel measurements of β-actin cDNA as an internal control.

### Flow Cytometry of HSC Progenitors

Bone marrow was harvested and single cell suspensions were prepared. Cell surface markers were adopted from previous publications which were defined and optimized for flow cytometry [25], [26]. Cells were biotin-labeled using a mouse hematopoietic lineage (Lin) flow panel (eBioscience #88–7774) and secondary labeled with Streptavidin-PE-Cy7 (eBioscience #25–4317). Specific fluorochrome-conjugated markers were APC anti-mouse CD117 (c-Kit, eBioscience #17–1171), Alexa Fluor anti-mouse Ly-6A/E (Sca-1, eBioscience #56–5981), eFluor 450 anti-mouse CD34 (eBioscience #48–0341), and Flk2-PE (BD #553842). Lineages were defined as long term hematopoietic stem cell (LT-HSC, Kit^+^; Sca^+^; Lin^-^; Flk2^-^; CD34^-^), short term hematopoietic stem cell (ST-HSC, Kit^+^; Sca^+^; Lin^-^; Flk2^-^; CD34^+^), multipotent progenitor (MPP, Kit^+^; Sca^+^; Lin^-^; Flk2^+^; CD34^+^), LSK (c-Kit^+^; Sca1^+^; Lin^–^, stem cell enriched population), common myeloid progenitor (CMP, Kit^+^; Sca^-^; Lin^-^; Flk2^+^; CD34^+^), common lymphoid progenitor (CLP, Kit^low^; Sca^low^; Lin^-^; Flk2^+^; CD34^+^), megakaryocyte-erythrocyte progenitor (MEP, Kit^+^; Sca^-^; Lin^low^; Flk2^+^; CD34^-^), and granulocyte-macrophage progenitor (GMP, Kit^+^; Sca^-^; Lin^low^; Flk2^+^; CD34^+^).

### Flow Cytometry of Splenic Lymphocytes

Single-cell suspensions were prepared from spleens using 70 µm sieves and erythrocytes were lysed. Cells were labeled with anti-CD45 APC (BD #559864), anti-B220 PE (BD #561878), and anti-CD3e FITC (BD #557666). Populations were defined as either T lymphocytes (CD45+; CD3+) or B lymphocytes (CD45+; B220+) using FACS Diva Software (3.0).

### Colony Forming Assays

For CFU-GEMM, CFU-GM, and BFU-E analysis, 2 × 10^4^ bone marrow cells were seeded in 35 mm dishes using methylcellulose-containing medium (STEMCELL Technologies) and colonies were scored 7–9 days later. For CFU-Meg analysis, 2 × 10^4^ bone marrow cells were seeded in 35 mm dishes using MegaCult-C medium (STEMCELL Technologies). After 8 days in culture, the semi-solid medium was dehydrated, fixed with acetone, and evaluated using acetylcholinesterase staining.

### Statistical Analysis

All results were expressed as means ± SEM. Statistical comparison of the different conditions was performed either by a pair wise *t* test or by an unpaired Student’s *t*-test. *, p<0.05; **, p<0.01.

## Results

### Tamoxifen-inducible Deletion of Jak2 during Mid/late Gestation Results in Hematopoietic Insufficiency and Death

Previous studies demonstrated that germline deletion of Jak2 results in embryonic lethality at E12.5 due to impaired hematopoiesis [8], [9]. The floxed Jak2 mice that we obtained for this current study were created independently of those previous two works. Specifically, the floxed Jak2 mice used here had loxP sites introduced around the ATG start codon in exon 2 [21]. Although it was shown that germline deletion of Jak2 in our mouse [21] recapitulates the previous two reports [8], [9], we first wanted to demonstrate that germline deletion of Jak2 in our hands would yield a similar result. We found that germline deletion of Jak2 derived from the Jak2 cKO mice in our hands resulted in a phenotype that was identical to the previous Jak2 conventional knockouts, as all our Jak2 null embryos were dead and partially resorbed at E13.5 and the reason for death was impaired hematopoiesis (Figure S1). As such, these results demonstrated a proof-of-concept in that they recapitulate the phenotype of the previously reported Jak2 conventional null embryos and therefore allowed for the deletion of Jak2 at later stages of prenatal and postnatal life.

To determine the role of Jak2 beyond germline deletion, we selected three different stages for Jak2 elimination; namely, mid/late gestation (E12.5), early postnatal life (PN4), or early adulthood (∼2 months of age). Figure 1A provides a summary of these optimized injection times and the days at which necropsy was performed for each specific cohort.

For the mid/late gestational experiments, timed matings were set with ROSA26^CreER/+^;*Jak2* ^f/f^ males and ROSA26^+/+^;*Jak2* ^f/f^ females. Tamoxifen (TM) was then injected into pregnant dams at 12.5 days post coitum (dpc). No ROSA26^CreER/+^;*Jak2* ^f/f^ newborn pups were found from the first several litters, indicating gestational lethality. To determine why lethality occurred in the Jak2 cKO mice, embryos from pregnant dams injected with TM at 12.5 dpc were examined at E17.5. All ROSA26^CreER/+^;*Jak2* ^f/f^ embryos (n = 13) were non-viable and resorbed at E17.5 when compared to controls (n = 20). The Jak2 conditional mutants were easily distinguished by pale yolk sacs and undersized embryos (Figure 1B). PCR analysis confirmed that 100% of these atypical yolk sacs were R26^CreER/+^;*Jak2* ^f/f^ (n = 13), and the null allele was only detected in these Jak2 mutant embryos (Figure 1B). In contrast, embryos containing the control genotype (ROSA26^+/+^;*Jak2* ^f/f^) appeared to be phenotypically normal (n = 20). The Jak2 mutant embryos themselves exhibited significant erythropoietic deficiency and umbilical vessels lacked red blood cells (Figure 1C). Spleens from the Jak2 cKO embryos were necrotic (Figure 1D). Compared to controls, the fetal livers from the Jak2 cKO embryos were smaller and had hematopoietic insufficiency characterized marked hypo-cellularity, reductions in erythroid and megakaryocytic precursors, and severe anemia (Figure 1E). Lastly, to determine the relative levels of expressed Jak2 in the control and Jak2 cKO embryos, sections of fetal liver were subjected to qRT-PCR mRNA analysis. We found that the Jak2 mRNA levels in the Jak2 cKO livers (n = 3) were <1% of that in controls (n = 3) (Figure 1F). These results indicate that the severe erythropoietic-deficiency phenotype observed in the Jak2 cKO embryos correlates with the virtual elimination of Jak2 from their livers.

In summary, the data in Figure 1 indicate that timed deletion of Jak2 beginning at mid-gestation (E12.5) results in lethality by E17.5, characterized by necrotic spleens and severely impaired erythropoiesis in the fetal liver.

### Tamoxifen-inducible Deletion of Jak2 during Early Postnatal Life Results in Death due to Severe Anemia

**Figure 2:**
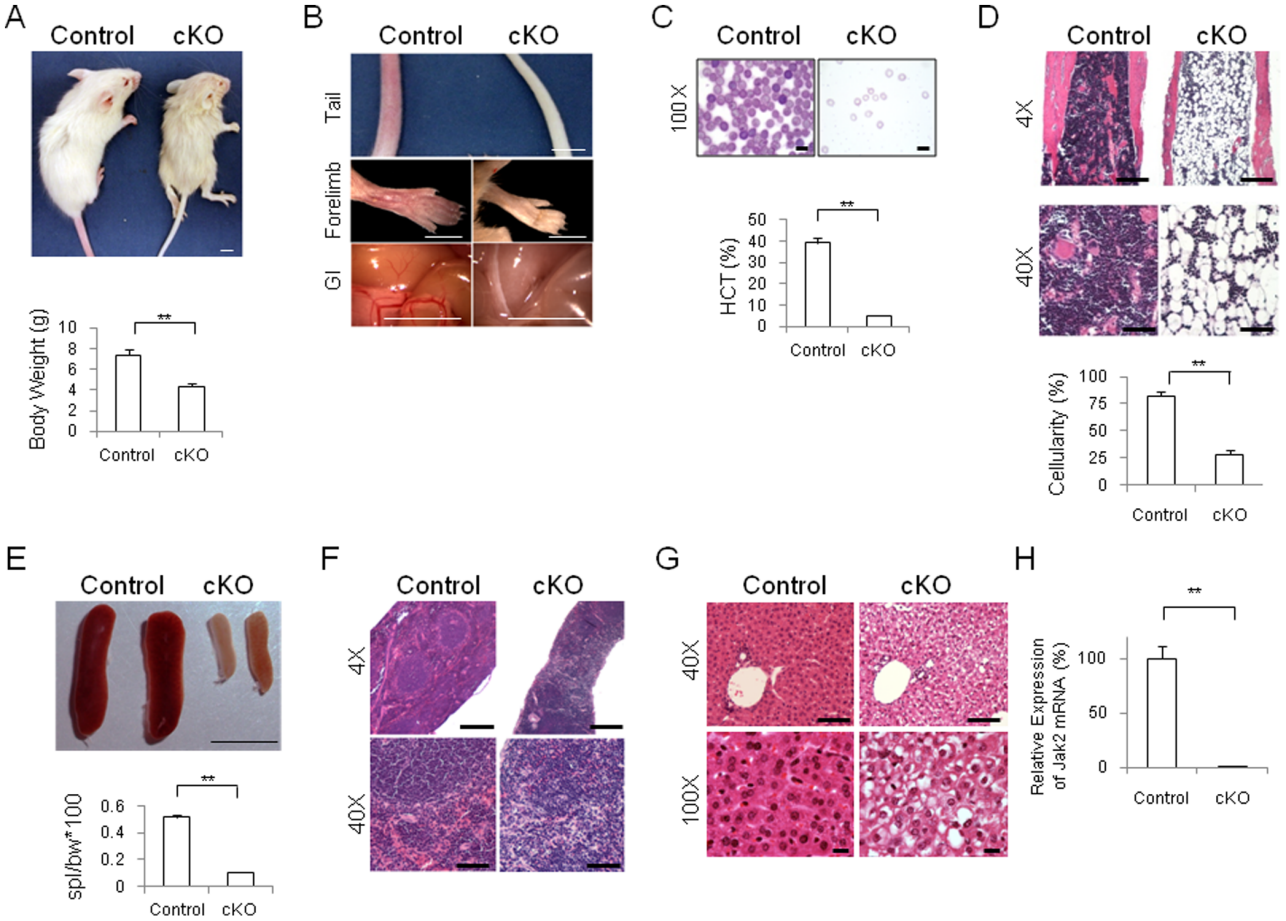
Deletion of Jak2 beginning at postnatal day 4 results in profound anemia. (A) Representative PN17 control (n = 7) and PN17 Jak2 cKO (n = 6) mice and body weights plotted as a function of genotype. Size bar  = 5 mm. (B) Pallor due to poor peripheral perfusion secondary to severe anemia was seen in the tail, forelimb, and mesenteric vasculature of the Jak2 cKO mice, but not the controls. Size bar  = 5 mm. (C) Representative peripheral blood films for both genotypes. Jak2 cKO blood films revealed marked anemia, hypochromasia, and microcytosis. Hematocrits plotted as a function of genotype indicating severe anemia in the Jak2 cKO mice. Size bars  = 10 µm. (D) Representative bone marrow sections from both genotypes and quantification of marrow cellularity plotted as a function of genotype. Size bars  = 500 µm for 4X images and 50 µm for 40X images. (E) Representative spleens from both genotypes and spleen weight to body weight ratios plotted as a function of genotype. Size bar  = 5 mm. (F) Histologic sections through the spleen are shown which indicate a hypoplasia phenotype in the Jak2 cKO mice. Size bars  = 500 µm for 4X images and 50 µm for 40X images. (G) Representative liver sections from both genotypes showing hepatocellular atrophy in the Jak2 cKO tissue, but not in the control tissue. Size bars  = 50 µm for 40X images and 10 µm for 100X images. (H) Determination of Jak2 mRNA levels in the liver from both genotypes. **, p<0.01.

To investigate the importance of Jak2 during early postnatal life, TM was administered to ROSA26^CreER/+^;*Jak2* ^f/f^ and ROSA26^+/+^;*Jak2* ^f/f^ mice beginning at postnatal day 4 (PN4). We found that all *Jak2* cKO mice were dead by PN25 (n = 18). Jak2 cKO mice administered TM at PN4 were clearly distinguishable from the control mice due to pallor and a reduced body size at PN17, and quantification of this size difference found it to be significant (Figure 2A). When compared to the controls, the tails, paws, and gastrointestinal system of the Jak2 cKO mice were very pale, suggestive of poor peripheral perfusion (Figure 2B). Analysis of the peripheral blood revealed significantly reduced numbers of platelets and marked microcytic hypochromic anemia and the hematocrits of the Jak2 cKO mice were reduced by ∼85%, when compared to controls (Figure 2C).

Characterization of the hematopoietic potential of these animals continued with analysis of the bone marrow, spleen, and liver. The Jak2 cKO marrow was markedly hypocellular and quantification of these data indicated that this difference was significant (Figure 2D). We found that the Jak2 cKO mice displayed an 80% reduction in spleen weight to body weight ratios when compared to controls (Figure 2E). Gross and histologic examination found that the Jak2 cKO spleens were hypoplastic, exhibited disorganized red and white pulp, and displayed erythroid extramedullary hematopoiesis (Figure 2F). Interestingly, analysis of splenic lymphocytes found that the percentage of lymphocytes in the Jak2 cKO mice were significantly elevated, when compared to controls (Table 1). Histologic examination of the liver revealed that the Jak2 cKO mice exhibited hepatocellular atrophy and lobular collapse when compared to controls (Figure 2G). Lastly, when compared to the controls, the levels of liver derived Jak2 mRNA were reduced by more than 99% in the Jak2 cKO mice (Figure 2H).

**Table 1:** Analysis of splenic lymphocytes at PN15 mice.

		CD45^+^B220^+^	CD45^+^CD3^+^	CD45^+^B220^+^CD3^+^	Total # ofsplenocytes
Absolute cell countper animal (×10^5^)	Control	29.4±1.3	8.9±2.0	7.4±1.1	142.3±7.1
	cKO	10.4±2.2	7.5±1.7	5.4±1.1	25.6±1.2
Number of eventsper 10^4^ total events	Control	2016±89	609±139	506±76	
	cKO	4072±863	2943±653	2099±412	
	Significance	p<0.01	p<0.01	p<0.01	

After TM injection at PN4, control (n = 3) and Jak2 cKO (n = 4) spleens were harvested at PN15 and subjected to flow cytometry for B cell (CD45+, B220+) and T cell (CD45+, CD3+) markers. Shown are the absolute numbers of cells per animal. Given that the total number of splenocytes are significantly reduced in the Jak2 cKO mice due to the smaller spleen sizes, the numbers of B cells and T cells are also shown after normalization per 10^4^ events.

Collectively, the data in Figure 2 and Table 1 indicate that loss of Jak2 beginning at PN4 results in animal death by PN25 via hematopoietic insufficiency and severe anemia.

### Tamoxifen-inducible Deletion of Jak2 Beginning in Early Adulthood Results in Impaired Hematopoiesis, but Higher Survival Rates

We next wanted to determine what effect, if any, the loss of functional Jak2 would have on adult animals. To gain some sense of how best to optimize Jak2 deletion in early adults, we used Rosa26-LacZ reporter mice, injected them with TM, and then examined β-gal expression patterns. This was an important issue as the data in Figures 1 and 2 showed that while TM is highly effective at deleting Jak2 (ie, >99%), however, it is not absolute. We found that a total of six TM injections for adult mice, comprised of three consecutive days of TM injections starting at PN35 and three consecutive days of TM booster injections starting at day 63, provided the highest level of β-gal expression when tissues were examined at PN91 (data not shown).

**Figure 3:**
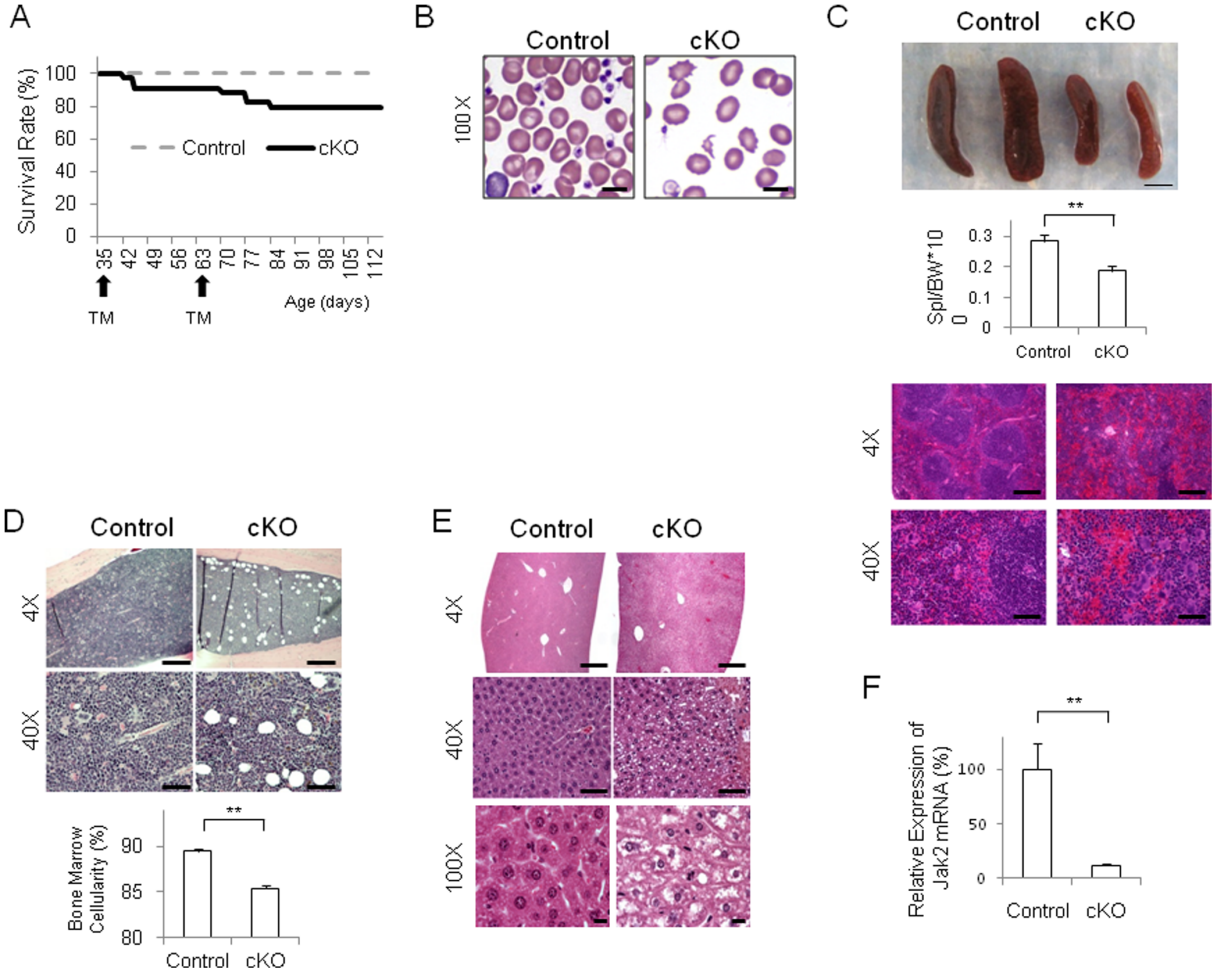
Tamoxifen-inducible deletion of Jak2 during early adulthood results in impaired hematopoiesis and lower mortality rates. (A) Kaplan-Meier survival curves for control (n = 17) and age-matched Jak2 cKO mice (n = 34). (B) Representative peripheral blood films from both genotypes showing the presence of anemia, thrombocytopenia, and poikilocytosis in the Jak2 cKO mice, but not the controls. Size bars  = 10 µm. (C) Representative spleens from both genotypes and spleen weight to body weight ratios plotted as a function of control (n = 10) and Jak2 cKO (n = 10) genotypes. Histologic sections of spleen found an abnormal splenic architecture in the Jak2 cKO mice characterized by atrophied and disorganized white pulp. Size bars  = 2 mm for top panel, 500 µm for 4X images and 50 µm for 40X images. (D) Representative bone marrow sections from both genotypes indicating increased adipose deposits in the Jak2 cKO mice, but not the controls. Also shown is bone marrow cellularity plotted as a function of control (n = 4) and Jak2 cKO (n = 5) genotypes. Size bars  = 500 µm for 4X images and 50 µm for 40X images. (E) Representative liver sections showing diffuse centrolobular vacuolar degeneration in the Jak2 cKO tissue, but not in the control tissue. Size bars  = 500 µm for 4X images, 50 µm for 40X images, and 10 µm for 100X images. (F) Determination of Jak2 mRNA levels from control (n = 4) and Jak2 cKO (n = 5) livers. **, p<0.01.

To determine the consequence of Jak2 deletion during early adulthood, control (ROSA26^+/+^;*Jak2* ^f/f^) and Jak2 cKO (ROSA26^CreER/+^;*Jak2* ^f/f^) mice were injected with TM following the optimized injection paradigm. Overall, we found that adult stage deletion of Jak2 resulted in a gross phenotype that was noticeably milder than that observed with the mid/late gestational and early postnatal Jak2 deletions. This was supported by the fact that death was observed in only 20% of the adult Jak2 cKO mutants, when compare to controls (Figure 3A). To gain a better understanding of the hematopoietic system within these animals, tissues were harvested at PN91 and analyzed. With respect to the peripheral blood, CBC and blood film evaluation indicated a number of abnormalities in the Jak2 cKO mice including significant reductions in red blood cells, hemoglobin, hematocrit, mean corpuscular hemoglobin, platelets, mean platelet volume, white blood cells, lymphocytes, monocytes, eosinophils, and basophils (Tables 2 and 3). The peripheral blood films from Jak2 cKO mice contained poikilocyte types and other morphologic changes including acanthocytes, schistocytes, echinocytes, elliptoechinocytes, spheroechinocytes, stomatocytes, and hypochromic microcytic erythrocytes (Figure 3B). Hemoglobin crystals were also qualitatively increased in Jak2 cKO mice, when compared to controls.

**Table 2:** CBCs from adult mice at Day 91.

	RBC	Hb	HCT	PLT	MCV	MCH	MCHC	RDW	MPV	PDW
	(M/µL)	(g/dL)	(%)	(K/µL)	(fL)	(pg)	(g/dL)	(%)	(fL)	(%)
Control	9.39±1.10	10.14±1.10	44.20±4.34	1058±43	47.24±3.18	10.81±0.53	22.93±0.98	20.59±2.05	3.94±0.35	31.44±3.51
cKO	5.86±1.43	5.67±1.14	25.50±4.26	292±21	44.47±5.24	9.79±0.60	22.12±1.45	18.20±2.73	3.40±0.39	29.54±3.62
% of Control	62.4%	55.9%	57.7%	27.6%	94.1%	90.5%	96.5%	88.4%	86.2%	90.4%
Significance	p<0.05	p<0.05	p<0.05	p<0.05	NS	p<0.05	NS	NS	p<0.05	NS

Shown are the absolute CBC values for control (n = 7) and Jak2 cKO (n = 7) mice and the values after they are normalized to control values. RBC (red blood cell), Hb (hemoglobin), HCT (hematocrit), PLT (platelet), MCV (mean corpuscular volume), MCH (mean corpuscular hemoglobin), MCHC (mean corpuscular hemoglobin concentration), RDW (red cell distribution width), MPV (mean platelet volume), and PDW (platelet distribution width). NS, not significant.

**Table 3:** CBCs from adult mice at Day 91.

	WBC	Neutrophils		Lymphocytes		Monocytes		Eosinophils		Basophils	
	(K/µL)	(K/µL)	(%)	(K/µL)	(%)	(K/µL)	(%)	(K/µL)	(%)	(K/µL)	(%)
Control	12.23±2.85	3.65±0.89	29.93±3.92	6.88±1.64	56.57±4.37	1.19±0.29	9.83±1.90	0.46±0.41	3.31±2.37	0.05±0.03	0.37±0.24
cKO	6.76±3.59	2.98±2.34	40.02±11.70	3.23±1.21	52.86±11.87	0.45±0.24	6.66±1.17	0.01±0.01	0.23±0.22	0.01±0.02	0.19±0.21
% of Control	55.3%	81.7%	133.7%	46.9%	93.5%	38.1%	67.8%	3.2%	7.1%	23.6%	52.3%
Significance	p<0.05	NS	NS	p<0.05	NS	p<0.05	p<0.05	p<0.05	p<0.05	p<0.05	NS

Shown are the absolute CBC values for control (n = 7) and Jak2 cKO (n = 7) mice and the values after they are normalized to control values. WBC (white blood cell). NS, not significant.

The spleen weight to body weight ratios were reduced by an average of 46% in the Jak2 cKO mice when compared to the controls and this was significant (Figure 3C). Histologic examination of this tissue revealed that Jak2 cKO spleens were comprised predominantly of red pulp with extramedullary hematopoiesis and had atrophied and disorganized white pulp (Figure 3C). The bone marrow of the Jak2 cKO mice was significantly less cellular, when compared to controls and this too was significant (Figure 3D). The Jak2 cKO livers exhibited diffuse centrilobular vacuolar degeneration consistent with hydropic change (Figure 3E). Lastly, we found that relative to controls, the levels of Jak2 mRNA in the Jak2 cKO mice at Day 91 were reduced by ∼88% (Figure 3F).

**Figure 4:**
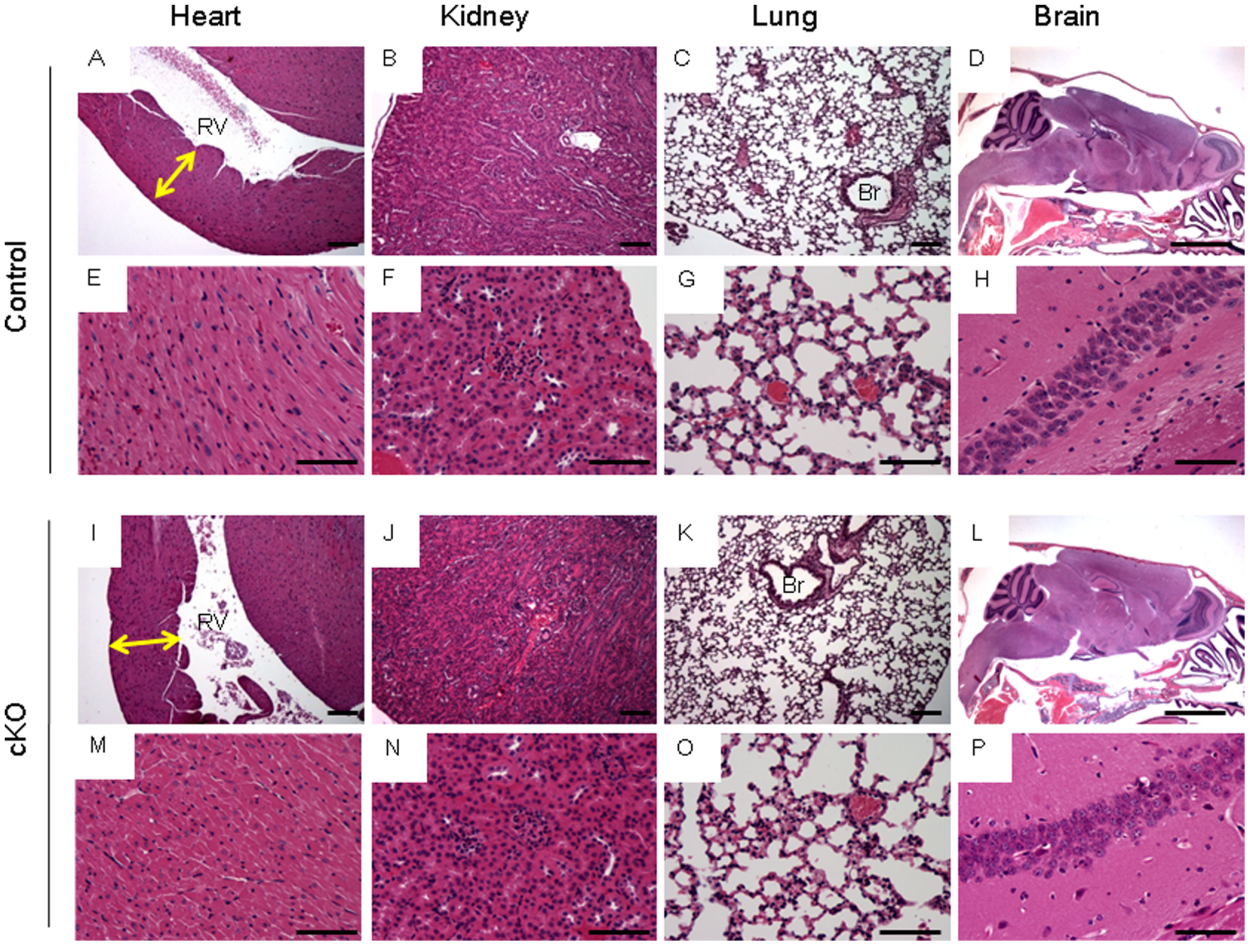
Histology of non-hematopoietic organs. Tissues were taken from Control (A-H) or Jak2 cKO (I-P) mice. Shown are representative hearts (A, E, I, and M), kidneys (B, F, J, and N), lungs (C, G, K, and O), and brains (D, H, L, and P). The relative magnifications are 4x (D and L), 10x (A–C and I–K), and 40x (E–H and M–P). For A and I, the yellow arrows indicate equal wall thickness and RV = right ventricle. For C and K, Br = bronchus. Size bars  = 500 µm (D&L), 100 µm (A–C, I–K), and 50 µm (E–H, M–P).

Jak2 is expressed in nearly every tissue in the body and it has been implicated in a number of other pathologies including renal injury, hypertension, and heart failure [20]. To determine what effect, if any, that deletion of Jak2 had on non-hematopoietic organs, we also examined heart, kidney, lung, and brain sections from these same TM treated animals (Figure 4). Overall, there was no marked difference in the histological appearance of these tissues between the two genotypes even though their hematopoietic systems were notably different.

Collectively, the data in Tables 2 and 3 as well as Figures 3 and 4 indicate that deletion of Jak2 in early adulthood results in abnormal hematopoiesis characterized by reduction in marrow cellularity, atrophied spleens, and reduced peripheral blood cell counts. This was coincident with a ∼88% reduction in Jak2 mRNA levels in the liver and a 20% death rate. Lastly, there were no marked differences in the histological appearance of the hearts, kidneys, lungs, and brains of these same TM treated animals.

### The Higher Survival Rate of the Adult Jak2 cKO Mice is due to Re-population of Hematopoietic Tissues with Jak2 Expressing Cells

**Figure 5:**
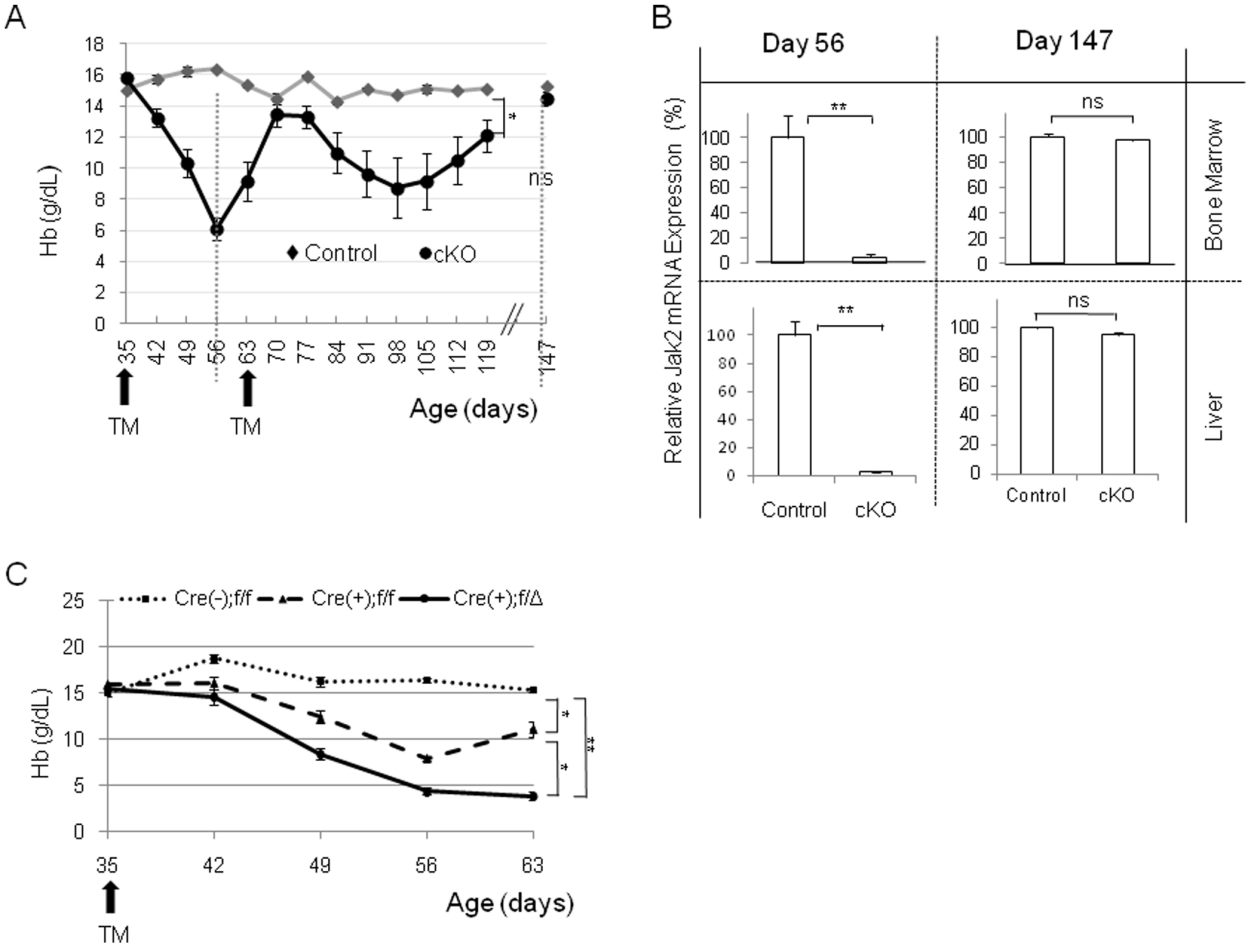
The higher survival rate observed in the adult Jak2 cKO mice is due to re-population of hematopoietic tissues with Jak2 expressing cells. (A) The levels of hemoglobin (Hb) plotted as a function of genotype and time. Although Hb levels dropped acutely in the Jak2 cKO mice (n = 7) relative to controls (n = 5) after TM injections, they returned to normal levels by day 147. (B) Shown are the relative levels of Jak2 mRNA in control (n = 3) and Jak2 cKO (n = 4) mice plotted as a function of day of collection, genotype, and tissue. (C) The levels of Hb plotted as a function of genotype and time. Shown are the control [Cre(-);f/f], Jak2 cKO [Cre(+);f/f], and the Jak2 compound mutant [Cre(+);f/Δ] genotypes. Conversion of one floxed Jak2 allele to a null allele [Cre(+);Jak2(f/Δ)] resulted in a marked and sustained reduction in Hb levels and ensuing death. *, p<0.05; **, p<0.01.

As opposed to the 100% lethality observed in the mid/late gestational and early postnatal deletions, deletion of Jak2 from young adult mice resulted in only a 20% death rate. One explanation for this was that TM was limited in its ability to delete Jak2 from the tissues. If this were the case, then over time, surviving Jak2 expressing cells could re-populate the hematopoietic tissues. An alternate explanation could be that in adulthood, there is functional redundancy by other proteins for the loss of Jak2 and this in turn allows for improved survival. To gain a better understanding of this process, mice of both genotypes were again subjected to the TM regimen at days PN35 and PN63. The erythropoietic state of the animals was then monitored longitudinally by measuring hemoglobin levels about once per week. As shown in Figure 5A, TM injection into the control mice was without effect. However, TM injection into the Jak2 cKO mice and subsequent Jak2 deletion resulted in cyclic erythropoiesis with the nadirs occurring around days 56 and 98. Interestingly, the hemoglobin levels returned to baseline levels by day 147. To understand what role Jak2 might play in this process, cohorts of mice were euthanized at days 56 and 147 so that the levels of Jak2 mRNA could be determined. At day 56, the levels of Jak2 mRNA in the Jak2 cKO mice were reduced by ∼95% in both the bone marrow and liver, when compared to control animals (Figure 5B). However, by day 147, the levels of Jak2 expression in the surviving Jak2 cKO mice were virtually the same as those of controls in both the liver and bone marrow (Figure 5B).

The data in Figs. 5A and 5B show that the levels of hemoglobin in the Jak2 cKO mice correlate positively with Jak2 expression levels in the liver and marrow. As such, these data suggest that the higher survival rate observed in adult animals is more likely due to repopulation of Jak2 expressing cells in hematopoietic tissues, rather than compensation by other proteins functioning in an environment that is devoid of Jak2. To demonstrate this experimentally, we created a compound mutant mouse that was comprised of one floxed and one null *Jak2* allele (f/Δ). The advantage of this mouse is that one Jak2 allele in each cell has already been deleted and hence, Cre recombinase only needs to delete the remaining Jak2 allele.

For this experiment, three genotypes were used; control (ROSA26^+/+^;*Jak2* ^f/f^), Jak2 cKO (ROSA26^CreER/+^;*Jak2* ^f/f^) and the newly created Jak2 compound mutant (ROSA26^CreER/+^;*Jak2* ^f/Δ^). Beginning on day 35, the mice received the TM regimen and hemoglobin levels were again determined weekly. Similar to Figure 5A, injection of TM into the control mice (ROSA26^+/+^;*Jak2* ^f/f^) had no effect on hemoglobin levels while TM injection into the Jak2 cKO mice (ROSA26^CreER/+^;*Jak2* ^f/f^) once again caused a significant decrease in the hemoglobin levels with the nadir occurring around day 56 and increasing by day 63 (Figure 5C). However, TM injection into the Jak2 compound mutants (ROSA26^CreER/+^;*Jak2* ^f/Δ^), resulted in an even greater and more sustained decrease in the hemoglobin levels and 100% mortality (n = 9) by day 72.

When taken together, the data in Figure 5 demonstrate that deletion of Jak2 in young adults temporarily reduces the hemoglobin levels to 6–8 g/dL and this is associated with a 20% death rate. At day 91, these animals have a number of hematopoietic abnormalities including peripheral blood cytopenias with abnormal erythrocyte morphology, decreased marrow cellularity, and splenic atrophy. However, the incomplete deletion of Jak2 by TM leaves some Jak2 expressing cells intact and the subsequent repopulation of the hematopoietic tissues by these cells normalizes the hemoglobin levels by day 147. When the f/Δ mice were injected with TM, there was a pronounced and sustained reduction (<5 g/dL for more than 7 days) of the hemoglobin concentrations and ensuing death. From this, we conclude that innate Jak2 actively restores hematopoietic homeostasis in the adult Jak2 cKO mouse, but that elimination of Jak2 via the combined effect of the null allele and TM-induced deletion of the floxed allele, results in death.

### Tamoxifen-inducible Deletion of Jak2 Significantly Attenuates *GBP-2* and *IRF-1* Expression at all three Deletion Time Points

**Figure 6:**
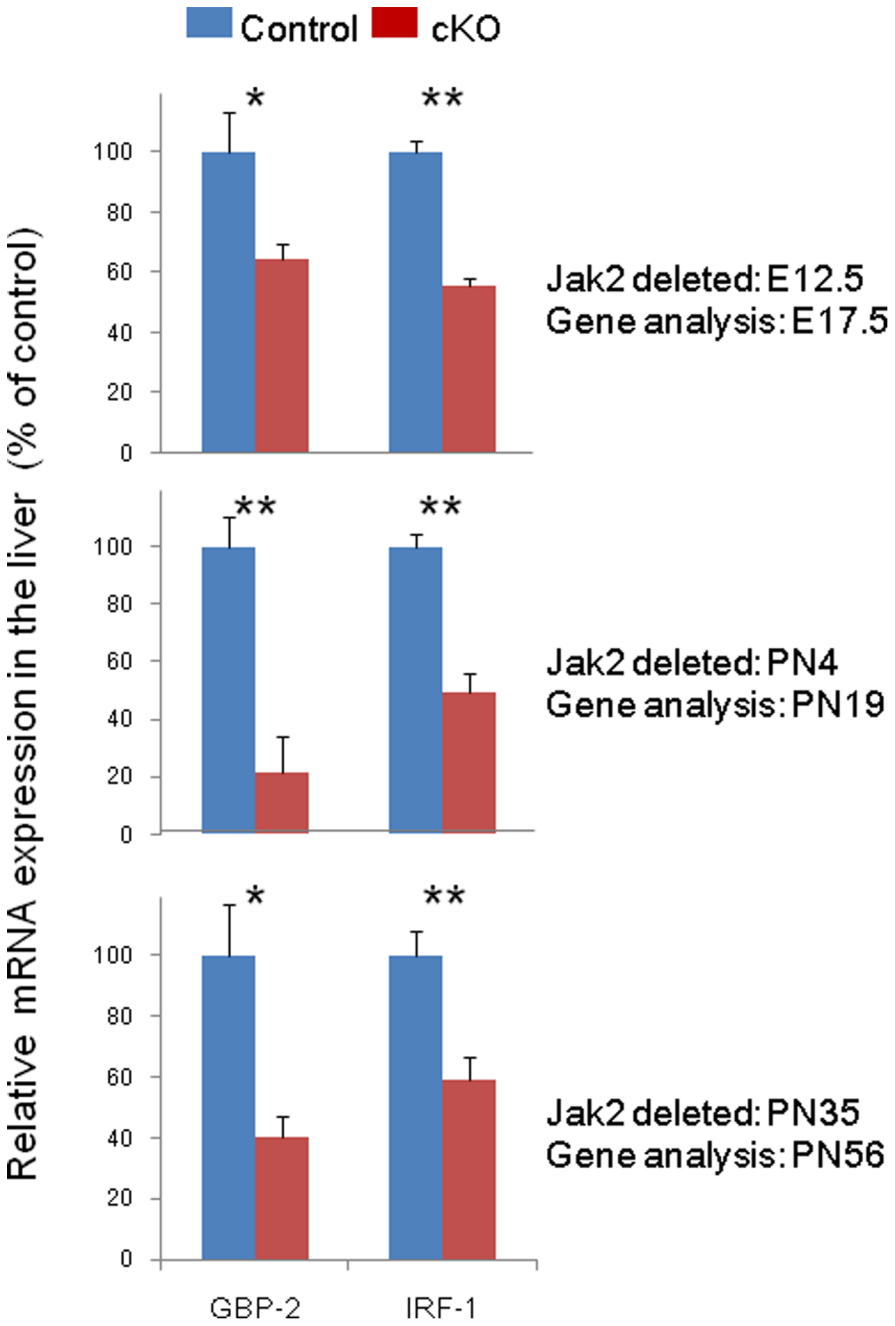
Tamoxifen-inducible deletion of Jak2 significantly attenuates *GBP-2* and *IRF-1* expression at all three time points. Shown are the relative levels of *GBP-2* and *IRF-1* mRNA in control and Jak2 cKO mouse livers plotted as a function of both time of Jak2 deletion and genotype. A total of 3–4 mice per genotype were used on each day and each sample was measured in triplicate. *, p<0.05; **, p<0.01.

Conditional deletion of Jak2 beginning at either E12.5, PN4, or PN35 results in marked hematopoietic defects characterized by a lack of definitive hematopoiesis/erythropoiesis (Figures 1, 2, 3). To demonstrate that these defects were consistent with a loss of signaling that is downstream of Jak2, we conducted gene profile analysis on two genes whose expression is known to be highly Jak2-dependent; namely, *GBP-2* and *IRF-1* [8], [9]. Here, Jak2 was conditionally deleted from mice via TM injection starting at either E12.5, PN4, or PN35 and livers were subsequently harvested at E17.5, PN19, and PN56, respectively. mRNA was extracted from the livers, reversed transcribed, and subjected to quantitative gene expression analysis. Relative to the control samples, we found that both *GBP-2* and *IRF-1* were significantly reduced in Jak2 cKO livers across all three time points, thereby confirming a functional loss of signaling that is known to be downstream of Jak2 (Figure 6). Furthermore, whereas the magnitude of the decreased expression of *IRF-*1 in the Jak2 cKO mice was similar across all three time points, the effect of Jak2 deletion on *GBP-2* expression in these same mice was greatest in early post natal life and least during late embryogenesis.

In summary, the data in Figure 6 indicate that deletion of Jak2 impacts Jak2-dependent downstream signaling as determined by the reduced levels of *GBP-2* and *IRF-1* mRNA in the livers of Jak2 cKO mice, relative to controls. Furthermore, the effect of Jak2 deletion on *IRF-1* expression was similar across the three time points whereas the effect of Jak2 deletion on *GBP-2* varied with the age of the animal.

### Tamoxifen-inducible Deletion of Jak2 during Early Adulthood Significantly Diminishes Myeloid, but not Lymphoid Progenitors

**Figure 7:**
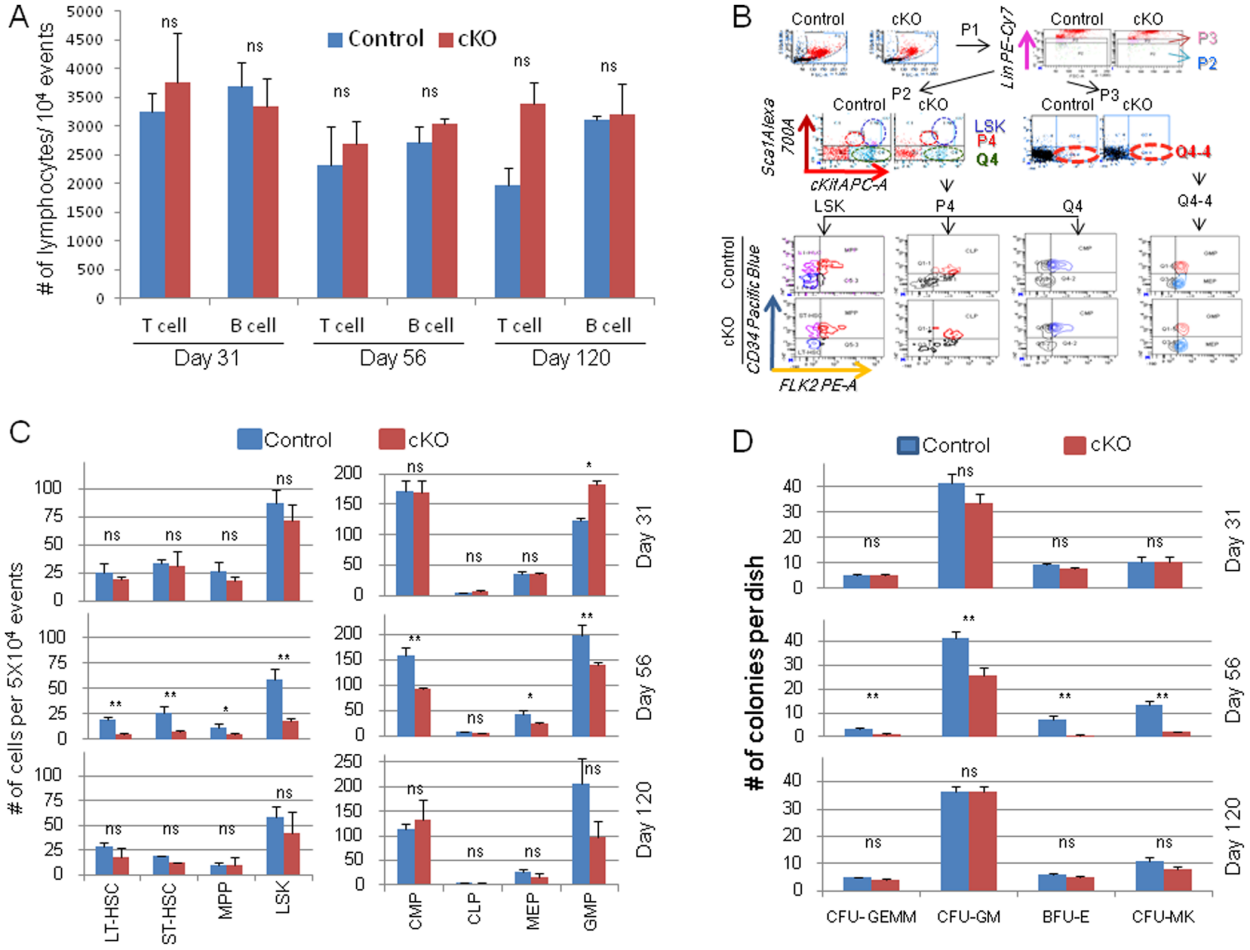
Tamoxifen-inducible deletion of Jak2 during early adulthood decreases stem cell and myeloid progenitors, but not lymphoid progenitors. (A) Spleens were harvested on the indicated days from the given genotypes and subjected to flow cytometry for analysis of T cell and B cell populations. Shown are the numbers of lymphocytes per 10^4^ total events. 3–5 mice of each genotype were used on each day. (B) Representative flow cytometry plots for Control and Jak2 cKO genotypes indicating the markers and gating that was employed for these studies. (C) The numbers of hematopoietic progenitors plotted as a function of genotype and day. Deletion of Jak2 in young adults diminished the numbers of LT-HSC, ST-HSC, MPP, LSK, CMP, MEP, and GMP, but not CLP. (D) Bone marrow cells were isolated on the indicated days from the given genotypes and cultured in semi-solid medium. The numbers of colonies were then determined and plotted as a function of both genotype and day. Relative to controls, the numbers of CFU-GEMM, CFU-GM, BFU-E, and CFU-MK were all significantly reduced in the Jak2 cKO mice at day 56, but not at days 31 and 120. A total of 3–4 mice per genotype were used on each day and each sample was grown in duplicate. *, p<0.05. **, p<0.01.

While the role of Jak2 on hematopoietic progenitor populations has been well defined in early embryogenesis [8], [9], the gestational lethality of Jak2 conventional knockout mice has precluded a similar examination in adult animals. Therefore, to determine the specific hematopoietic lineages that were impacted by Jak2 deletion in the adult mouse, we repeated the longitudinal study shown in Figure 5A whereby mice received the TM dosing regimen starting on day 35 and cohorts of mice were sacrificed either at baseline (Day 31), at the hematopoietic nadir (Day 56), or at recovery (Day 120). Within the spleen, we found no significant differences in the number of T cells and B cells at any of the three adult time points (Figure 7A). We then examined the numbers of hematopoietic progenitors within the bone marrow. Figure 7B displays representative flow cytometry plots at Day 56 while Figure 7C indicates the aggregate numbers of progenitors plotted as a function of both genotype and day. Overall, we found no significant decreases in any hematopoietic progenitors either at baseline (Day 31) or at recovery (Day 120). However, significant decreases were observed in the Jak2 cKO mice at the hematopoietic nadir (Day 56). Specifically, at Day 56, when compared to controls, the Jak2 cKO mice had significantly reduced numbers of LT-HSC, ST-HSC, MPP, LSK, CMP, MEP, and GMP, but not CLP.

To determine if the reduced numbers of stem and myeloid progenitors impacted the clonogenic growth potential of these cells, bone marrow cells were also plated in semi-solid media and the number of colony forming units were determined. We observed no significant differences between the two genotypes in the clonogenic growth potential for any of the hematopoietic progenitors collected either on Day 31 or on Day 120 (Figure 7D). However, at Day 56, Jak2 cKO had significantly reduced numbers of CFU-GEMM (3.0±0.59 vs. 1.25±0.2), CFU-GM (41.3±3.0 vs. 24.3±3.2), BFU-E (7.0±1.8 vs. 0.8±0.3), and CFU-MK (13.3±1.6 vs. 2.0±0.4), when compared to controls. Interestingly, although all myeloid lineages were significantly reduced in the Jak2 cKO cells when compared to the controls, there appeared to be a differential effect of Jak2 on myelopoiesis as deletion of Jak2 severely impaired erythropoiesis and thrombopoiesis, but only had a mild to moderate effect on granulopoiesis and monocytopoiesis (Figure 7D).

Collectively, the data in Figure 7 indicate that deletion of Jak2 in the adult mouse diminishes myeloid, but not lymphoid progenitors. Furthermore, within the myeloid compartment, deletion of Jak2 in young adults severely impaired erythropoiesis and thrombopoiesis more so than granulopoiesis and monocytopoiesis. As such, Jak2 appears to have a specific yet critical effect on hematopoiesis in young adult animals.

## Discussion

Hematopoiesis is a complex process involving scores of cytokines and dozens of kinases. At least ten different *Src* family kinases and all four *Janus* family kinases are expressed within hematopoietic cells [1]–[4]. In the case of Src family kinases, they are important for a number of hematopoietic responses including pro T cell and pro B cell development, megakaryocytopoiesis, thrombopoiesis, erythroblast expansion, and myeloproliferation [27]–[31]. In the case of Jak2, the germline deletion demonstrated the critical role of Jak2 in the establishment of the embryonic hematopoietic system [8], [9]. However, the embryonic death of those mice precluded determination of the role of Jak2 in maintaining hematopoiesis in postnatal animals. Our results here demonstrate that Jak2 plays a similarly critical role in maintaining hematopoiesis in neonatal and adult animals. Further, the results show that there is no compensation by other kinases for the functional loss of Jak2 in adults. As such, we conclude that Jak2 plays a critical and non-redundant role in hematopoiesis during prenatal and postnatal life.

Jak2 is ubiquitously expressed. Not surprisingly, it has been implicated in a number of pathologies including some of the heart, kidneys, lungs, and brain [32]–[35]. Analysis of these non-hematopoietic tissues from our adult mice found no marked gross or histological difference between the two genotypes. However, there were marked differences in the hematopoietic tissues that were harvested from these same animals. The implication of our results is that chronic Jak2 inhibition or absence of appreciable Jak2 function will impact the hematopoietic systems before these others.

**Figure 8:**
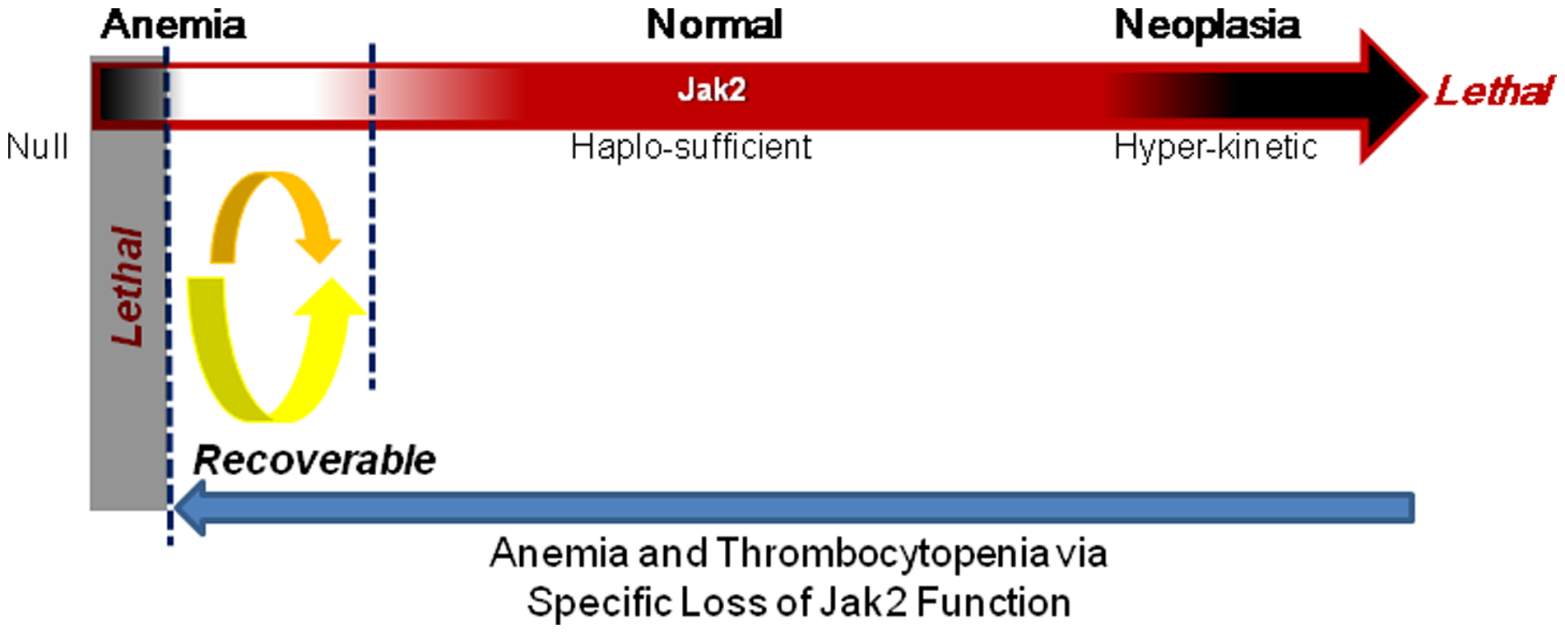
Proposed role of Jak2 in adult hematopoiesis. Hyper-kinetic Jak2 signaling results in various neoplastic disorders in adult animals and in some instances, this can be lethal. On the other hand, the specific loss of Jak2 function in adults results in anemia and thrombocytopenia. The severity of these disorders is directly proportional to the extent to which Jak2 function is lost. For instance, an 85%-95% decrease in Jak2 mRNA levels is coincident with significantly decreased bone marrow cellularity, atrophied spleens, and reduced peripheral blood cell counts. Yet, there is still a potential to recover from this pathogenic state. However, when Jak2 mRNA levels are reduced by >95% (ie, Jak2 is eliminated from >95% of cell), there is concomitant thrombocytopenia, severe anemia, and eventual death.

We previously showed that adult mice that have only one functional Jak2 allele (ie, a 50% reduction in Jak2) are phenotypically normal [36]. Given that the Cre-mediated excision of Jak2 from the genome is an all or nothing event, in this current work, we used Jak2 mRNA levels as a surrogate for populations of cells. We found that an 88% reduction in Jak2 mRNA levels (ie, ∼88% of cells experienced Cre-mediated Jak2 deletion) in the adult mice was associated with abnormal hematopoiesis characterized by reductions in marrow cellularity, atrophied spleens, reduced peripheral blood cell counts, and 20% mortality. In the adult Jak2 compound mutant mice where one allele was null and the other was deleted with TM (ROSA26^CreER/+;^Jak2^f/Δ^), we found that there was a >95% decrease in Jak2 mRNA levels in the marrow and liver when compared to controls (ROSA26^+/+^;Jak2^f/f^), and 100% mortality characterized by a severe lack of erythropoiesis. Thus, we have defined relative threshold levels of Jak2 that are needed to maintain hematopoietic viability within adult animals and described the associated phenotype that is observed when Jak2 function *per se*, is increasing lost (Figure 8).

Another important aspect of this work is the creation and characterization of the mice described herein. Specifically, the Jak2 cKO mice (ROSA26^CreER/+^;Jak2^f/f^) are a powerful tool for exploring the role of Jak2 in physiology and disease. In our case, we used the inducibility of Cre expression to escape the embryonic lethality of conventional Jak2 deletion and thereby defined specific roles for Jak2 in post natal life. Furthermore, primary cells can be obtained from the bone marrow or other tissues of these mice, cultured *ex vivo*, and Jak2 can then be deleted from the cells via the addition of TM to the culture media. This approach provides for a more precise inhibition of Jak2 when compared to Jak2 pharmacological inhibitors and unlike Jak2 siRNA, the down regulation/deletion of Jak2 within a given cell is permanent. That said, there are also limitations with these mice. For example, as noted earlier, a significant amount of time was spent identifying a TM injection protocol that would allow for the highest degree of Cre expression in all cells of an adult animal. Despite this optimization and subsequent 95% reductions of Jak2 mRNA levels in the bone marrow and liver, there was eventual re-population of hematopoietic tissues with Jak2 expressing cells. As such, the Jak2 compound mutant mice (ROSA26^CreER/+^;*Jak2* ^f/Δ^) also described in this work should be used in combination with the cKO mice. Lastly, while our studies have defined novel roles for Jak2 in adult animals, they cannot distinguish between potential non-autonomous functions of Jak2. In other words, given that the deletion of Jak2 was from virtually every cell type in the mouse, it is possible that a supporting cell type (ie, stromal cell), may be providing the critical requirement for Jak2 function in hematopoiesis. However, given that the flow cytometry and clonogenic growth potential assays were done using individual hematopoietic cells, this is unlikely. That said, current studies which are utilizing allogenic bone marrow transplants between Jak2 cKO mice and littermate controls will help to resolve this issue.

Given the inability of first generation Jak2 inhibitors to provide marked bone marrow efficacy in the form of histopathologic, cytogenetic, or molecular remission [15]–[18], the overall impact of Jak2 inhibition on the bone marrow is not fully understood. In addition, it has recently been argued that more Jak2 inhibitors need to be developed and studied in order to not only identify more efficacious drugs, but to determine the consequence of long term Jak2 inhibition in animals [19], [37]. In one regard, the TM-inducible system that we employed here is a case of extreme Jak2 inhibition. Rather than causing enzymatic inhibition of the protein, TM causes permanent deletion of Jak2 from genetically modified cells. Our results here demonstrate that virtual elimination of wild type Jak2 activity can ultimately lead to severe anemia/thrombocytopenia and even death. At the same time however, the ability of the few surviving Jak2 clones to subsequently re-populate hematopoietic tissues underscores the challenges of permanently ridding the bone marrow of targeted clones. Our results also appear to explain adverse events that were noted in a recent human study. Specifically, when the Jak2 inhibitor Ruxolitinib/Jakafi was administered to myelofibrosis patients with the intent of reducing blood transfusion dependency via the targeted suppression of mutant granulocyte/macrophage progenitors, it was reported that the drug was more likely to cause anemia and thrombocytopenia, than to correct them [18]. This is consistent with our observations whereby deletion of Jak2 in our adult animals virtually eliminated erythrocyte and thrombocyte growth potentials, but had only a moderate effect on ganulocyte/macrophage potentials. As such, Jak2 inhibitors may be more suited for the treatment of polycythemia vera and/or essential thrombocythemia, diseases which are characterized by expanded erythrocyte and thrombocyte lineages, respectively. Given that there are clinical trials which are currently evaluating the use of Jak2 inhibitors for the treatment of these diseases, data will soon become available that will either support or refute this hypothesis. In another example of how the observations in our mice have therapeutic relevance, it was previously reported that the Jak2 inhibitor, SB1518, was effective at suppressing the growth of both myeloid and lymphoid malignancies [38]. Given our results here indicating normal lymphopoiesis in the Jak2 cKO mice, we conclude that the suppression of lymphoid malignancies by SB1518 is occurring via a mechanism that is independent of Jak2 inhibition. Consistent with this is the observation that SB1518 inhibits Tyk2 and FLT3 kinases with a potency that is similar to Jak2 [38] and thus, suppression of lymphoid malignancies by SB1518 may occur via the inhibition of one or both of these enzymes.

In summary, the loss of functional Jak2 at three different stages of mouse ontogeny results in hematopoietic insufficiency and death. From these results, we conclude that Jak2 plays a critical and non-redundant role in hematopoiesis during both prenatal and postnatal life. Furthermore, delineation of the hematopoietic lineages that are sensitive to the loss of Jak2 function in an adult animal has relevance to current attempts to inhibit Jak2 kinase function for the treatment of human diseases.

## Supporting Information

Figure S1 **Germline derived Jak2 conditional knockout mice recapitulate the Jak2 null phenotype.** (A) Yolk sacs, embryos, and embryo sections at E12.5 of control (Jak2^f/f^) and germline derived Jak2 cKO mice (Jak2^Δ/Δ^). The Jak2 cKO embryos were pale due to lack of red blood cells. The green arrowheads mark the fetal livers and the lack of red blood cells in the Jak2^Δ/Δ^ embryo. Sagittal sections of whole embryos identified an abnormal liver (blue arrowheads) and heart (red arrowheads) in the Jak2 cKO mice. Size bars  = 1 mm. (B) Representative liver sections of both genotypes showing marked hypo-cellularity in the Jak2 null embryo. Size bars  = 50 µm (C) Genotyping of the floxed and null Jak2 alleles by genomic PCR analysis.
(TIF)
