# Supplementary figures and images for: Conditional Deletion of Jak2 Reveals an Essential Role in Hematopoiesis throughout Mouse Ontogeny: Implications for Jak2 Inhibition in Humans
Source: PLoS One. 2013 Mar 27;8(3):e59675. doi: 10.1371/journal.pone.0059675 (PMC3609865; doi:10.1371/journal.pone.0059675)

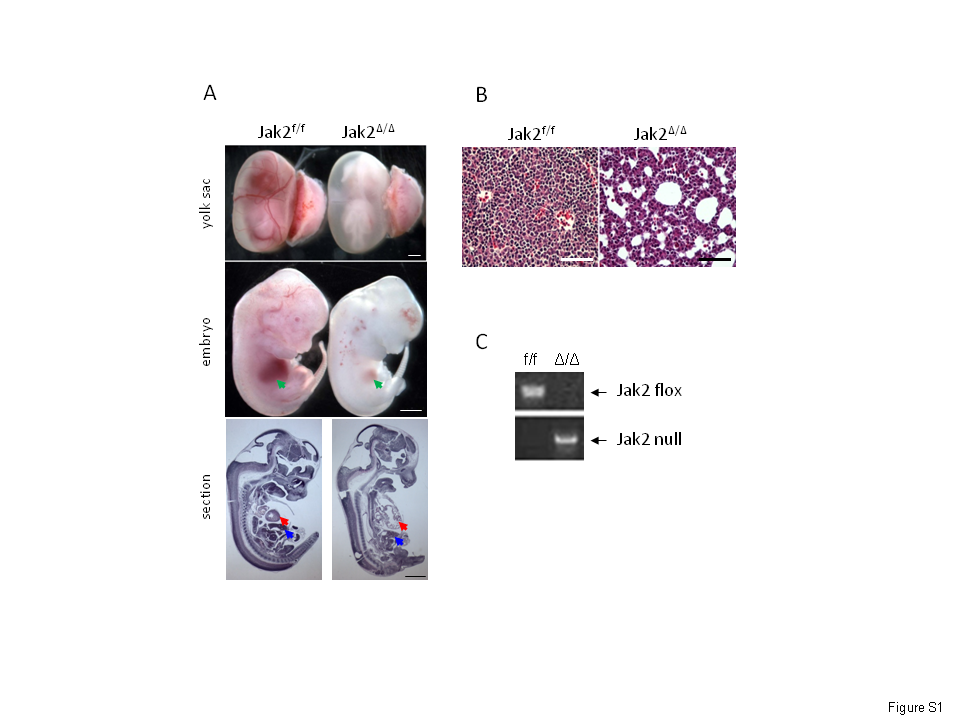

Supplement: Figure S1 — Germline derived Jak2 conditional knockout mice recapitulate the Jak2 null phenotype. (A) Yolk sacs, embryos, and embryo sections at E12.5 of control (Jak2f/f) and germline derived Jak2 cKO mice (Jak2Δ/Δ). The Jak2 cKO embryos were pale due to lack of red blood cells. The green arrowheads mark the fetal livers and the lack of red blood cells in the Jak2Δ/Δ embryo. Sagittal sections of whole embryos identified an abnormal liver (blue arrowheads) and heart (red arrowheads) in the Jak2 cKO mice. Size bars = 1 mm. (B) Representative liver sections of both genotypes showing marked hypo-cellularity in the Jak2 null embryo. Size bars = 50 µm (C) Genotyping of the floxed and null Jak2 alleles by genomic PCR analysis. (TIF) [file pone.0059675.s001.tif]
